# Comparative long-term outcomes of kidney transplantation from donors with isolated microscopic hematuria and thin basement membrane disease versus donors with isolated microscopic hematuria and normal histology: the largest cohort analysis to date

**DOI:** 10.3389/frtra.2026.1952224

**Published:** 2026-10-08

**Authors:** Yaser Shah, Dieter Broering, Dalia Obeid, Hassan Aleid, Ahmed Nazmi, Hani Alahdal, Kris Ann Hervera Marquez, Turki AlHussain, Tariq Ali

**Affiliations:** 1 Department of Organ Transplant Centre of Excellence (OTCoE), King Faisal Specialist Hospital and Research Centre, Riyadh, Saudi Arabia; 2 Department of Pathology, King Faisal Specialist Hospital and research centre, Riyadh, Saudi Arabia

**Keywords:** allograft survival, donor biopsy, graft outcomes, kidney transplantation, living kidney donor, thin basement membrane disease (TBMD)

## Abstract

**Background:**

Thin basement membrane disease (TBMD) represents a frequent etiology of isolated microscopic hematuria (IMH) and is often identified during the evaluation of living kidney donor candidates. Although typically regarded as a benign condition, its genotypic overlap with Alport spectrum disorders has raised concerns regarding potential post-transplant implications. This study aimed to compare long-term graft and recipient outcomes following kidney transplantation from donors with biopsy-confirmed TBMD versus donors exhibiting IMH with normal histopathological findings.

**Methods:**

A retrospective cohort analysis was conducted including 93 living donor–recipient pairs: 39 donors (41.9%) with TBMD and 54 donors (58.1%) with normal biopsy results despite IMH. Donor and recipient demographic, clinical, and immunologic parameters were examined. Primary outcomes encompassed renal function, persistence of hematuria, proteinuria, arterial blood pressure, biopsy-proven rejection episodes, and both patient and graft survival. Follow-up duration extended up to 9 years (median 3 years).

**Results:**

Baseline demographic and clinical characteristics were comparable between the two groups. At 12 months post-transplant, mean serum creatinine levels did not differ significantly between recipients of TBMD donor kidneys and those of normal biopsy donors (95 µmol/L vs. 88.5 µmol/L; *p* = 0.579). Renal function and graft survival remained stable over the follow-up period. The incidences of delayed graft function and biopsy-confirmed acute rejection were low and similar between groups. Persistent microscopic hematuria was observed in 87.2% of recipients from TBMD donors and 73.1% of those from normal biopsy donors; however, none developed albuminuria. One-year graft survival was excellent and sustained at the latest follow-up (97.4% vs. 98.1%).

**Conclusions:**

Kidney allografts from donors with TBMD demonstrate long-term graft function and survival outcomes equivalent to those from donors with IMH and normal histology, supporting the overall safety of the allograft after kidney transplantation from TBMD donors. Although persistent microscopic hematuria was common, it did not adversely affect graft outcomes. Further large-scale, prospective, and genetically stratified studies including both recipients and donors are warranted to confirm long-term allograft safety and to refine donor selection criteria.

## Introduction

Isolated microscopic hematuria (IMH) is a relatively common finding, with reported prevalence ranging from approximately 1% to 20% in the general population, depending on multiple factors including age, sex, screening methods, diagnostic criteria, and differences among studies from various geographic regions (1, 2). In regions like the Middle East, and specifically Saudi Arabia, living donation heavily utilizes familial networks, significantly raising the clinical stakes regarding heritable glomerular conditions. Thin basement membrane disease (TBMD) is a common cause of persistent IMH, with hallmark histological findings of diffuse thinning of the glomerular basement membrane (GBM) (3). In a previous study by our group, approximately 45% of potential kidney donors with IMH were found to have TBMD on renal biopsy, suggesting that this finding is not uncommon among individuals with IMH (4). TBMD is usually considered a benign condition, particularly in the absence of extrarenal, syndromic features or a family history suggestive of hereditary nephritis. Nevertheless, TBMD exists on a spectrum with Alport syndrome, both linked to mutations in COL4A3 and COL4A4, raising legitimate concerns about the potential for progression to proteinuria, declining kidney function, or adverse transplant outcomes in the recipient (4–6).

These concerns are particularly relevant in the context of kidney transplantation, where the long-term safety of using kidneys from donors with subclinical glomerular abnormalities, such as TBMD or persistent IMH, remains a subject of ongoing debate. A previous small study involving 11 recipients of kidneys from donors with biopsy-confirmed TBMD reported favorable midterm outcomes, including preserved graft function and minimal complications during up to five years of follow-up (7). Conversely, other studies have shown that up to 15% of patients with TBMD in the native kidneys may develop proteinuria, and a subset may even progress to nephrotic syndrome (8). However, interpretation of these outcomes is constrained by the limited sample size, the inherent difficulty in differentiating early Alport syndrome from benign TBMD, and the incomplete characterization of the prevalence and persistence of hematuria or proteinuria in recipients after kidney transplantation (4, 5, 7, 9).

Additionally, direct evidence regarding recipient outcomes following transplantation from donors with IMH but normal biopsy findings remains limited. Moreover, comparative investigations assessing outcomes among recipients of kidneys from biopsy-confirmed TBMD donors versus those from donors with IMH and normal biopsy findings—are virtually absent from the literature. The sensitivity of pre-donation urine microscopy for detecting underlying glomerular pathology appears moderate at best, underscoring the diagnostic value of kidney biopsy prior to living donation in cases of persistent IMH (4, 9).

Given these knowledge gaps, our study represents the largest reported cohort to date evaluating recipient outcomes following living donor kidney transplantation from donors with either biopsy-proven TBMD or IMH with normal kidney histology. We focus on clinically relevant endpoints, including longitudinal graft function, persistence of hematuria, and the development of proteinuria. The findings from this study are expected to provide meaningful evidence to guide future guidelines and clinical decision-making regarding the safety and appropriateness of accepting kidneys from these donor phenotypes. Therefore, this study aimed to determine the outcomes of kidney transplant recipients who received kidneys from donors with TBMD and to compare them with those who received kidneys from donors with IMH but normal kidney biopsy findings.

## Material and methods

This retrospective cohort study included all kidney transplant recipients who received allografts from living donors with either biopsy-confirmed thin basement membrane disease (TBMD) or normal histologic findings in the context of persistent isolated microscopic hematuria (IMH). The study period extended from November 2015 to February 2024. Ethical approval was obtained from the institutional Research Ethics Committee as part of the authorized use of retrospective registry data (RAC No. 2121012), and all procedures were conducted in accordance with the principles of the Declaration of Istanbul and the Declaration of Helsinki.

All potential living kidney donors underwent comprehensive clinical and laboratory evaluations in accordance with institutional protocols and international donor selection standards (4, 10). Urinalysis was performed on at least two separate occasions to confirm the persistence of microscopic hematuria, using both dipstick testing and microscopic examination of urinary sediment (4). Albumin and total protein excretion were measured to assess for urinary protein loss, and renal function was evaluated using serum creatinine levels and cystatin C–based estimated glomerular filtration rate (eGFR). All donors underwent CT imaging to assess renal anatomy and exclude structural abnormalities. Donors demonstrating reduced eGFR, albuminuria, proteinuria, or structural abnormalities were deemed ineligible for donation and were also excluded from the study.

Patients with clinical features suggestive of Alport syndrome, including sensorineural hearing loss, anterior lenticonus, or a family history of hereditary nephritis, were excluded prior to biopsy. Donors with persistent IMH and no exclusion criteria underwent kidney biopsy in accordance with standard institutional protocols. Histopathologic evaluation included standard tri-modal assessment: light microscopy (including hematoxylin and eosin, Jones silver, and periodic acid–Schiff staining), panel-wide immunofluorescence (staining for IgG, IgA, IgM, C3, and C1q) to systematically rule out IgA nephropathy or other immune complex-mediated lesions, and transmission electron microscopy (TEM). All biopsy specimens were reviewed by our expert renal transplant pathologists in collaboration with the transplant nephrology team. A diagnosis of TBMD was established when electron microscopy demonstrated diffuse thinning of the glomerular basement membrane, defined as a mean thickness below 250 nm. To completely avoid tissue shrinkage artifacts associated with the recovery of paraffin-embedded material, ultrastructural evaluation was performed using fresh cortical tissue immediately fixed in 2.5% glutaraldehyde, post-fixed in 1% osmium tetroxide, and embedded in epoxy resin (Epon). Pathologists utilized the digital (simple) measurement method across three distinct glomerular cross-sections. Perpendicular cross-sectional lines were drawn from the outer endothelial cell membrane to the base of the podocyte plasma membrane across a minimum of 20 randomly selected peripheral capillary loops per glomerulus to determine the baseline arithmetic mean and standard deviation.

Donors with either normal biopsy findings or biopsy-confirmed TBMD were deemed eligible for kidney donation. Recipients were subsequently categorized into two groups according to the histopathological findings of their respective donors: the **TBMD group**, comprising recipients of grafts from donors with biopsy-proven thin basement membrane disease, and the **Normal Biopsy group**, comprising recipients of grafts from donors with normal histologic findings.

Demographic and clinical data for both donors and recipients were obtained from the institutional transplant registry and hospital electronic medical records. Collected variables included recipient and donor age, sex, cause of end-stage renal disease (ESRD), donor–recipient relationship, and results of human leukocyte antigen (HLA) typing and crossmatch testing.

Induction immunosuppression was administered in accordance with departmental protocols: thymoglobulin was used for recipients at high immunological risk, basiliximab for those at low immunological risk, and no induction therapy for fully HLA-matched pairs.

Clinical outcomes assessed included serum creatinine levels at predefined post-transplant time points, blood pressure measurements during follow-up visits, and urine albumin-to-creatinine ratios. Delayed graft function, as well as patient and graft survival, were also evaluated. Episodes of acute rejection were documented and defined according to the Banff criteria (11, 12).

## Satistical analysis

All statistical analyses were conducted using R software version 4.5.1 (R Foundation for Statistical Computing) within the RStudio environment. Descriptive statistics were used to summarize baseline demographic and clinical characteristics. Continuous variables were compared using either the Welch's *t*-test or the Wilcoxon rank-sum test, depending on the distribution of the data as assessed for normality. Categorical variables were compared using the chi-square test or Fisher's exact test, as appropriate. A two-sided *P*-value of <0.05 was considered statistically significant.

Survival analysis was performed using the Kaplan–Meier method, with differences between groups assessed using the log-rank test. Overall survival was defined as the time from transplantation to either graft failure or patient death. Cox proportional hazards models were used to estimate hazard ratios (HRs) with corresponding 95% confidence intervals (CIs) in both univariate and multivariable analyses. Outcomes of interest included graft failure and a composite endpoint of death or graft failure (referred to as the “worst outcome”).

## Results

### Baseline characteristics

Baseline clinical and demographic characteristics of the study cohort are presented in Table 1. The study included 93 living donor–recipient kidney transplant pairs, comprising recipients who received kidneys from donors diagnosed with thin basement membrane disease (TBMD) (*n* = 39, 41.9%) or from donors with normal biopsy findings (*n* = 54, 58.1%). Recipient follow-up ranged from 1 to 9 years, with a median duration of 3 years. The mean ages of donors and recipients were 37 and 39 years, respectively, and the majority of recipients were male (64.5%). Nearly half of the recipients (47.3%) had an unknown underlying cause of kidney disease at the time of transplantation.

**Table 1:** Baseline characteristics of kidney transplant recipients/donors by thin basement membrane disease (TBMD) presence in donors.

Characteristic	Overall (*N* = 93)	Normal Biopsy (*N* = 54)	TBMD (*N* = 39)	*p*-value
Donor age, years	36.9 (30.6–42.3)	32.4 (28.4–41.6)	41.0 (34.5–43.6)	<0.001
Male donor	60 (64.5)	40 (74.1)	20 (51.3)	0.023
Donor relation to recipient				0.003
Sibling	35 (37.6)	20 (37.0)	15 (38.5)	
Paired kidney exchange (PKE)	19 (20.4)	13 (24.1)	6 (15.4)	
Child	14 (15.1)	13 (24.1)	1 (2.6)	
Parent	13 (14.0)	3 (5.6)	10 (25.6)	
Other^a^	12 (12.9)	5 (9.3)	7 (18.0)	
Recipient is an adult	82 (88.2)	51 (94.4)	31 (79.5)	0.047
Recipient age, years	39 (27–54)	40 (28–58)	37 (20–46)	0.048
Male recipient	58 (62.4)	35 (64.8)	23 (59.0)	0.566
Primary diagnosis				0.319
Unknown	44 (47.3)	25 (46.3)	19 (48.7)	
Glomerulonephritis (GN)	14 (15.1)	7 (13.0)	7 (18.0)	
Diabetes mellitus (DM)	13 (14.0)	11 (20.4)	2 (5.1)	
Hypertension (HTN)	12 (12.9)	5 (9.3)	7 (18.0)	
Other	7 (7.5)	4 (7.4)	3 (7.7)	
Urological	3 (3.2)	2 (3.7)	1 (2.6)	
Transplant number				>0.99
First	85 (91.4)	49 (90.7)	36 (92.3)	
Second	8 (8.6)	5 (9.3)	3 (7.7)	
HLA mismatch groups				0.989
0–3	50 (53.8)	29 (53.7)	21 (53.9)	
4–6	43 (46.2)	25 (46.3)	18 (46.2)	
Crossmatch				0.136
Negative	89 (95.7)	50 (92.6)	39 (100.0)	
Positive	4 (4.3)	4 (7.4)	0 (0.0)	
Induction therapy				0.553
ATG	62 (66.7)	38 (70.4)	24 (61.5)	
Basiliximab	26 (28.0)	14 (25.9)	12 (30.8)	
None	5 (5.4)	2 (3.7)	3 (7.7)	

Values are median (IQR) for continuous variables and *n* (%) for categorical variables.

*p*-values from Welch's *t* test, Pearson's *χ*^2^ test, Fisher's exact test, or Wilcoxon rank-sum test, as appropriate.

TBMD, thin basement membrane disease; PKE, paired kidney exchange; DM, diabetes mellitus; GN, glomerulonephritis; HTN, hypertension; HLA, human leukocyte antigen, ATG, Anti-Thymocyte Globulin.

a Other: include spouse, other relative and non-relative donors.

Donors with TBMD were significantly older than those with normal biopsy findings (mean age: 41.0 vs. 32.4 years; *p* < 0.001). Conversely, recipients who received kidneys from TBMD donors were slightly younger than those who received kidneys from donors with normal biopsy findings (mean age: 37.0 vs. 40.0 years; *p* = 0.048).

Histopathologic evaluation of baseline architecture confirmed high-quality, parenchymal tissue across all accepted donor kidneys. Chronic parenchymal changes were uniformly minimal or absent and global glomerulosclerosis was safely <5% in both donor cohorts. Furthermore, 100% of the included biopsies demonstrated completely negative or non-specific trace background staining on panel-wide immunofluorescence, confirming the successful exclusion of subclinical IgA nephropathy.

Siblings constituted the largest group of donors (37.6%), followed by participants in paired kidney exchange programs (20.4%). Other donor–recipient relationships included children donating to parents (15.1%), parents donating to children (14.0%), and other relatives or unrelated donors (12.9%). Most recipients were undergoing their first kidney transplantation (91.4%), while a minority had received a previous transplant (8.6%).

Baseline immunologic characteristics were comparable between the two groups, with no significant differences in HLA mismatches or induction immunosuppressive therapy.

A summary of the demographic and clinical characteristics of the donor-recipient pairs, stratified by TBMD status, is provided in Table 1.

### Recipient outcomes

At 12 months post-transplantation, the mean serum creatinine levels were comparable between recipients of kidneys from donors with TBMD and those from donors with normal biopsy findings, measuring 95 µmol/L and 88.5 µmol/L, respectively (*p* = 0.579). Similarly, no significant difference in mean serum creatinine levels was observed between the groups at the last follow-up (Table 2). Blood pressure (BP) remained well controlled in both groups throughout the follow-up period. At the final visit, systolic and diastolic BP readings were comparable between the groups (*p* = 0.454 and *p* = 0.137, respectively). The incidence of delayed graft function and biopsy-proven acute rejection within the first year post-transplant was low and did not differ significantly between the two groups (Table 2).

**Table 2:** Kidney transplant recipients’ outcomes by thin basement membrane disease (TBMD) presence in donors.

Characteristic	Overall (*N* = 93)	Normal Biopsy (*N* = 54)	TBMD (*N* = 39)	*p*-value
Serum creatinine, μmol/L				
Day 5	123.5 (86–182.5)	131.0 (86–179)	119.0 (86–202)	0.628
Day 30	101.5 (80–123)	95.0 (76–111)	110.0 (84–128)	0.929
3 months	96.0 (79–117)	94.5 (78.5–111)	100.5 (80–122)	0.860
6 months	92.0 (78–116)	91.5 (77–111)	98.0 (78–116)	0.954
12 months	90.0 (75.5–113)	88.5 (75–113)	95.0 (78–115)	0.579
Last systolic BP, mmHg	124 (116–135)	125 (117–135)	123 (116–138)	0.454
Last diastolic BP, mmHg	79 (72–83)	77 (72–82)	81 (75–86)	0.137
Last serum creatinine, μmol/L	89.5 (73–110)	88.5 (72.5–113)	91 (78–105)	0.759
Last urine ACR, mg/mmol	1.4 (0.9–3.3)	1.1 (0.8–2.6)	2.2 (1.0–5.1)	0.141
Hematuria (recipient)				0.101
Yes	72 (79.1)	38 (73.1)	34 (87.2)	
No	19 (20.9)	14 (26.9)	5 (12.8)	
Recipient alive	90 (96.8)	51 (94.4)	39 (100.0)	0.262
Delayed graft function	4 (4.3)	2 (3.7)	2 (5.1)	>0.99
Graft functioning	91 (97.9)	53 (98.1)	38 (97.4)	>0.99
Biopsy at 1 year	26 (28.0)	14 (25.9)	12 (30.8)	0.608
Acute rejection at 1 year	12 (12.90)	6 (11.11)	6 (15.38)	0.544
Banff category (*n* = 12)				0.697
Borderline	8 (66.7)	3 (50.0)	5 (83.3)	
1A	2 (16.7)	1 (16.7)	1 (16.7)	
1B	1 (8.3)	1 (16.7)	0 (0.0)	
2B	1 (8.3)	1 (16.7)	0 (0.0)	

Values are median (IQR) for continuous variables and *n* (%) for categorical variables.

*p*-values from Welch's *t* test, Pearson's *χ*^2^ test, Fisher's exact test, or Wilcoxon rank-sum test, as appropriate.

TBMD, thin basement membrane disease; ATG, anti-thymocyte globulin; BP**,** blood pressure; ACR, albumin-to-creatinine ratio.

Persistent isolated microscopic hematuria (IMH) was observed in the majority of recipients, occurring in 87.2% of the TBMD group and 73.1% of the Normal group. Importantly, none of the recipients in either group developed albuminuria during the follow-up period (Table 2 and Figure 1).

**Figure 1:**
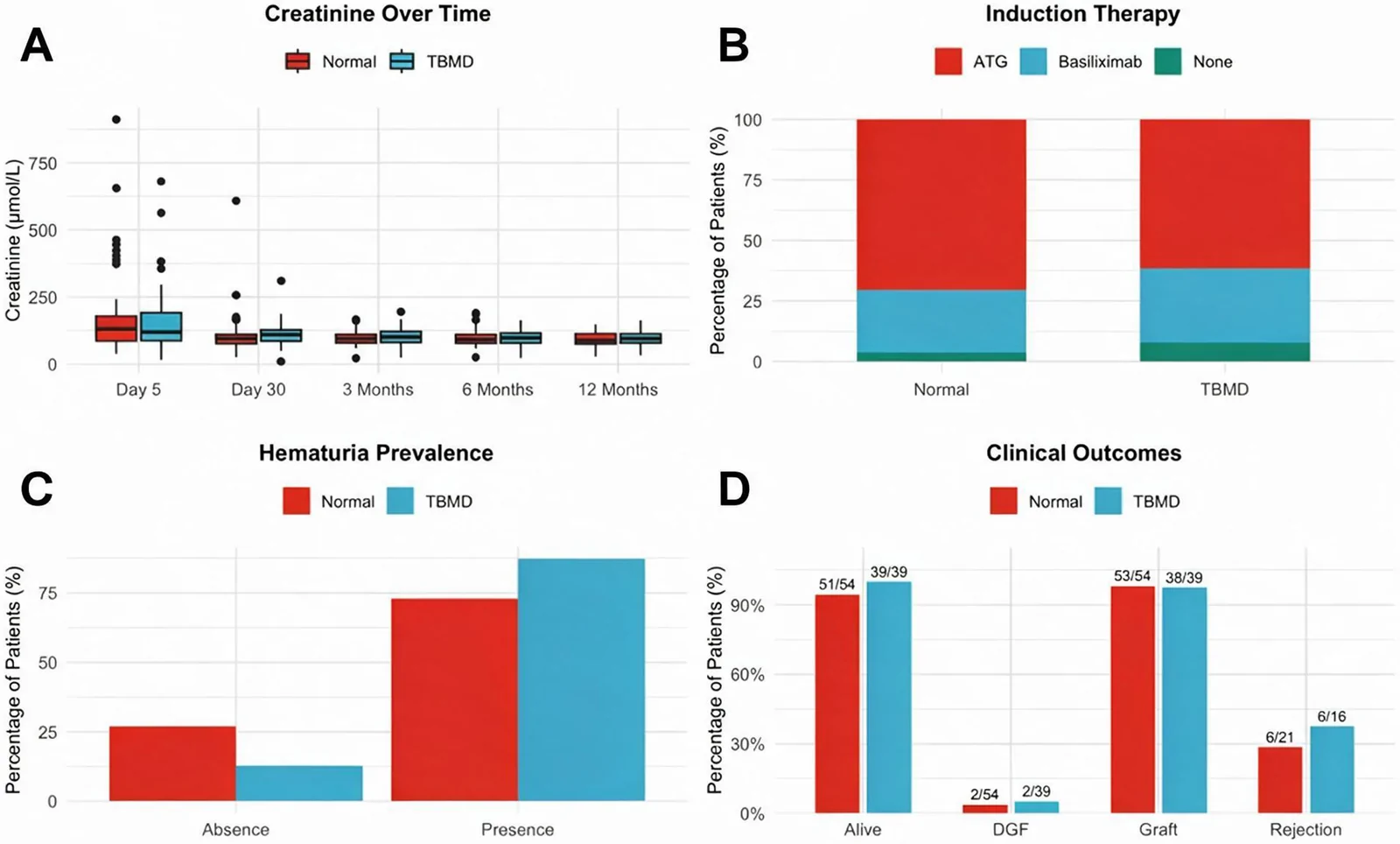
Recipent's outcomes by thin basement membrane disease (TBMD) status. **(A)** shows the serum creatinine levels (µmol/L) over time after transplantation (day 5, day 30, 3 months, 6 months, and 12 months) in recipients of kidneys from donors with normal histology (red) or TBMD (blue). Values are displayed as box plots, with horizontal lines representing the medians and whiskers representing the interquartile range. **(B)** depicts the distribution of induction therapy used at the time of transplantation in each group. **(C)** shows the prevalence of hematuria during the follow-up period. **(D)** Bar plot summarizes the major clinical outcomes, including patient survival (alive), delayed graft function (DGF), graft survival, and acute rejection within the first year after transplantation. Bars indicate the percentage of patients, with counts displayed above each bar.

Despite the persistence of IMH, graft outcomes were excellent in both groups, with one-year graft survival rates of 97.4% in the TBMD group and 98.1% in the Normal biopsy groups respectively (Table 3; Figures 2 A,B).

**Table 3:** Kaplan–meier estimates for 3 years graft survival/worst outcome of recipients of kidneys from TBMD vs normal donors.

Outcome	Group	6 mo	12 mo	18 mo	24 mo	30 mo	36 mo
Worst Outcome	Normal	0.96 (0.91–1.00)	0.94 (0.89–1.00)	0.93 (0.86–1.00)	0.93 (0.86–1.00)	0.93 (0.86–1.00)	0.93 (0.86–1.00)
	TBMD	0.97 (0.93–1.00)	0.97 (0.93–1.00)	0.97 (0.93–1.00)	0.97 (0.93–1.00)	0.97 (0.93–1.00)	0.97 (0.93–1.00)
	*P*-value*	0.32	–	–	–	–	–
Graft Failure	Normal	0.98 (0.95–1.00)	0.98 (0.95–1.00)	0.98 (0.95–1.00)	0.98 (0.95–1.00)	0.98 (0.95–1.00)	0.98 (0.95–1.00)
	TBMD	0.97 (0.93–1.00)	0.97 (0.93–1.00)	0.97 (0.93–1.00)	0.97 (0.93–1.00)	0.97 (0.93–1.00)	0.97 (0.93–1.00)
	*P*-value*	0.82	–	–	–	–	–

(*) Log rank test.

**Figure 2:**
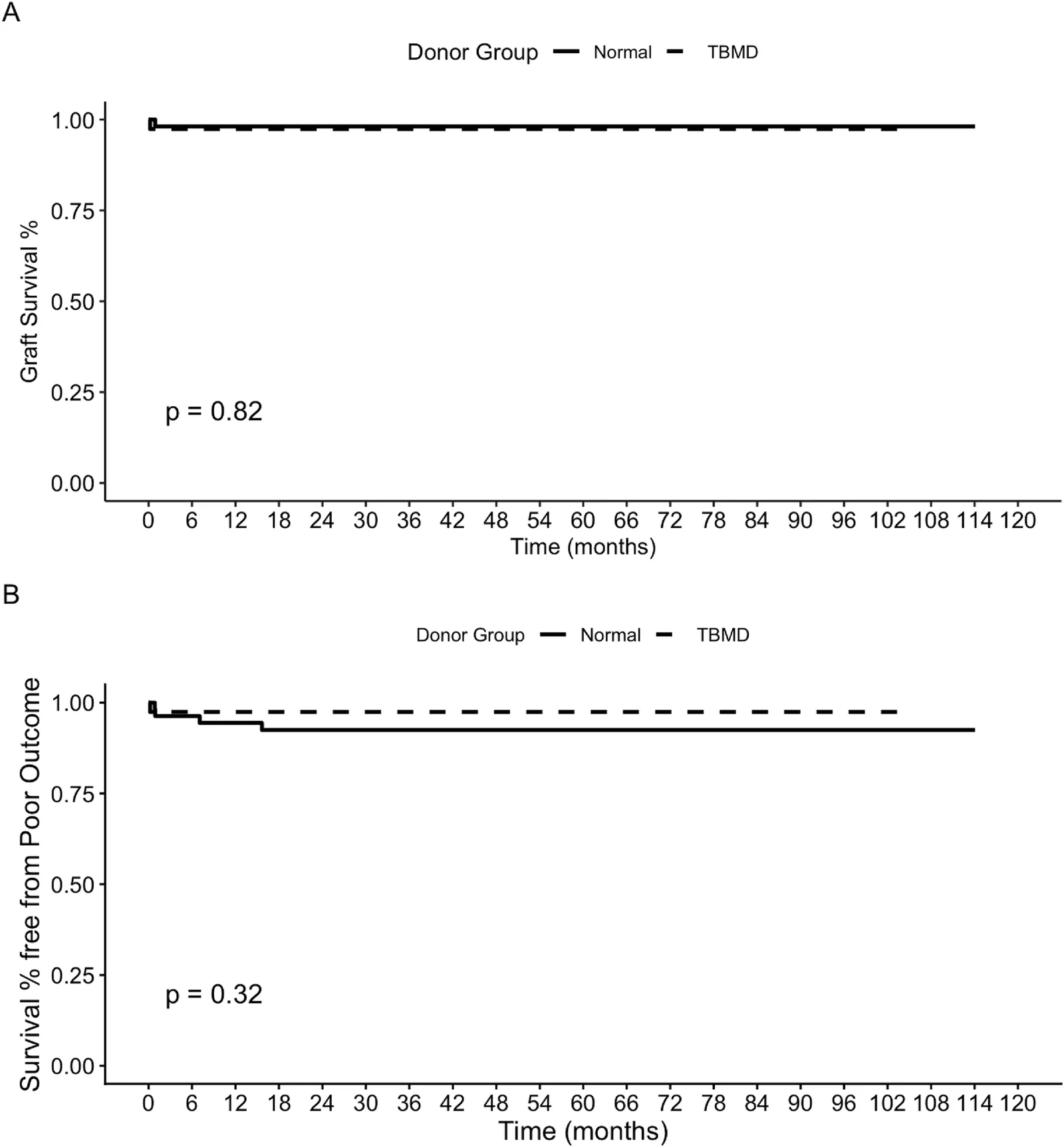
**(A)** Kaplan–Meier Estimates of Graft Survival by Donor’ Histology. Kaplan–Meier curves showing graft survival among recipients of kidneys from donors with normal histology (solid line) and from donors with TBMD (dashed line). No significant difference was observed between the two groups (*P* = 0.82, log-rank test). **(B)** Kaplan–Meier Estimates of worst outcomes by Donors’ Histology. Kaplan–Meier curves showing survival among recipients of kidneys free of worst outcomes (graft failure and death) from donors with normal histology (solid line) and from donors with TBMD (dashed line). No significant difference was observed between the two groups (*P* = 0.32, log-rank test).

Univariate and multivariate analyses were performed to identify predictors of graft failure and adverse outcomes among recipients in both groups. None of the donor or recipient factors—including age, sex, and HLA mismatches—were found to have a significant impact on graft outcomes (Tables 4, 5).

**Table 4:** Multivarite and univarite model predicting graft faillure using cox regression.

Characteristic	Univariate		Multivariate	
	HR (95% CI)	*p*-value	HR (95% CI)	*p*-value
TBMD Vs Normal (Ref)	1.39 (0.09 to 22.2)	0.82	1.37 (0.06 to 31.1)	0.84
Female Vs Male Donors	1.79 (0.11 to 28.6)	0.68	1.37 (0.06 to 29.6)	0.84
Donor Age	1.16 (0.96 to 1.41)	0.13	1.23 (0.93 to 1.63)	0.14
Recipient Age	1.03 (0.96 to 1.12)	0.40	1.06 (0.97 to 1.16)	0.21
Female Vs Male recipient	1.67 (0.10 to 26.6)	0.72	4.35 (0.14 to 133)	0.40
HLA Groups				
(4−6) Vs (0−3)	1.16 (0.07 to 18.6)	0.91	0.82 (0.04 to 16.7)	0.90

CI, Confidence Interval; HR, Hazard Ratio.

**Table 5:** Multivarite and univarite model predicting worst outcome using cox regression.

Characteristic	Univariate		Multivariate	
	HR (95% CI)	*p*-value	HR (95% CI)	*p*-value
TBMD Vs Normal (Ref)	0.35 (0.04 to 3.12)	0.35	0.20 (0.02 to 2.21)	0.19
Female Vs Male Donors	2.70 (0.45 to 16.2)	0.28	3.83 (0.57 to 25.9)	0.17
Donor Age	1.09 (0.98 to 1.21)	0.13	1.09 (0.98 to 1.20)	0.1
Recipient Age	1.01 (0.96 to 1.06)	0.72	1.00 (0.95 to 1.06)	0.98
Female Vs Male recipient	0.41 (0.05 to 3.65)	0.42	0.53 (0.05 to 5.27)	0.59
HLA Groups				
(4–6) Vs (0–3)	1.76 (0.29 to 10.6)	0.53	1.83 (0.28 to 12.0)	0.53

CI, Confidence Interval; HR, Hazard Ratio.

### Sensitivity analysis by donor relationship type

To evaluate the potential clinical impact of heritable traits and high-risk genetic constellations as shown in Table 6, a sub-group sensitivity analysis was performed stratifying the 93 donor-recipient pairs into a High Familial Risk group (siblings, parents, and children; *n* = 62) and a Low/Unrelated Risk group (paired kidney exchange, spouses, and unrelated donors; *n* = 31). At 12 months post-transplantation, median serum creatinine levels did not differ significantly between the familial and unrelated groups (92.5 μmol/L vs. 88.0 μmol/L, respectively; *p* = 0.375). Furthermore, there were no statistically significant differences in the prevalence of persistent microscopic hematuria in recipients (80.6% vs. 71.0%; *p* = 0.301) or biopsy-proven acute rejection rates within the first year (14.5% vs. 9.7%; *p* = 0.751). Kaplan–Meier estimates confirmed equivalent long-term graft survival trajectories across both genetic relationship strata (log-rank *p* = 0.612), showing that our rigorous clinical screening and tri-modal biopsy protocol safely mitigated immediate heritable risks.

**Table 6:** Recipient clinical outcomes stratified by donor genetic relatedness.

Clinical Outcome Parameter	High Familial Risk (*n* = 62)	Low/Unrelated Risk (*n* = 31)	Statistical Test	*p*-value
Median 12-Month Creatinine (*μ*mol/L)	92.5	88.0	Wilcoxon Rank-Sum	0.375
Persistent Recipient Hematuria, *n* (%)	50 (80.6%)	22 (71.0%)	Fisher's Exact Test	0.301
Biopsy-Proven Rejection at 1 Year, *n* (%)	9 (14.5%)	3 (9.7%)	Fisher's Exact Test	0.751
36-Month Graft Survival Rate (%)	98.4%	96.8%	Log-Rank Test	0.612

## Discussion

Our study demonstrates that the outcomes of kidney transplant recipients who received kidneys from donors with thin basement membrane disease (TBMD) are excellent and comparable to those from donors with normal kidney histology. These findings may provide reassurance to patients and healthcare professionals regarding the acceptability of using kidneys from donors with TBMD. To the best of our knowledge, this is the first study to directly compare transplant outcomes between TBMD donors and donors with normal kidney histology.

Thin basement membrane disease (TBMD), also referred to as thin basement membrane nephropathy, is a genetically influenced renal disorder characterized by diffuse thinning of the glomerular basement membrane (GBM), most frequently presenting with persistent microscopic hematuria and generally following a benign clinical course (13). The studies have reported that the incidence of TBMD is about 1% in the general population (14). While the majority of TBMD cases are indolent and maintain stable kidney function throughout life, a subset of patients may progress to end-stage renal disease, necessitating kidney replacement therapy, including transplantation (6, 15). A noteworthy case report described a presumed monozygotic twin who donated a kidney to her twin sister (16). Preimplantation biopsy of the donor kidney revealed TBMD. Although the etiology of end-stage renal disease in the recipient was uncertain, it may have been related to TBMD. Nevertheless, both the donor and the recipient demonstrated favorable short-term outcomes (16). The selection of kidney allografts from donors with TBMD has historically raised concerns regarding the potential for adverse recipient outcomes, such as anti-GBM nephritis or graft rejection, due to histopathological similarities with Alport syndrome (17); however, published series demonstrate that these complications are uncommon in TBMD allografts and graft function is maintained during short- to midterm follow-up (7). While historical literature has cited lower TBMD thresholds ranging from 191 nm to 200 nm, our choice of a 250 nm cutoff aligns directly with contemporary international nephropathology standards for adult populations. In the highly sensitive scenario of living kidney donor selection, utilizing a conservative 250 nm threshold prevents the over-acceptance of donors with subtle, early-stage basement membrane attenuation, thereby protecting the living donor from post-nephrectomy hyperfiltration injury on an already compromised structural network.

Another concern occasionally raised pertains to kidney paired donation (KPD). In KPD programs, maintaining balanced exchanges between donor–recipient pairs are debated to ensure equity and comparable graft quality (18). A potential challenge arises when one donor is found to have TBMD on biopsy, while the paired donor shows no features to suggest underlying histologic abnormalities. This discrepancy may raise concerns regarding fairness and equivalence in organ quality between the paired recipients. However, our findings indicate that transplant outcomes from donors with TBMD are excellent and comparable to those from donors with normal histology. These results provide reassurance that kidneys from donors with TBMD can be considered acceptable for inclusion in paired exchange programs.

Our study represents the largest published cohort to date reporting the outcomes of kidney transplant recipients who received organs from donors with biopsy-proven TBMD, as well as those from donors with IMH and normal biopsy findings. Previous work, notably by Choi et al., described 11 living-donor transplants wherein donor kidneys manifested TBMD on pretransplant biopsy; follow-up protocol biopsies in recipients revealed normal GBM thickness in the majority, raising the possibility of sampling variability and a mosaic distribution of TBMD within the renal parenchyma (7, 17). This transient nature of GBM thinning which disappeared post-transplant calls into question the reliability of TBMD diagnosis in the previous study (7).

In the present study, all pretransplant donor biopsies were evaluated by our experienced renal pathologists in collaboration with a transplant nephrologist. Donor acceptance required comprehensive clinical assessment and exclusion of Alport syndrome features or a suggestive family history.

Interestingly, Choi et al. reported resolution of microscopic hematuria in their cohort (7). In contrast, microscopic hematuria persisted in almost 87% of recipients in our cohort with TBMD. Despite this finding, all recipients maintained excellent graft function and remained free of albuminuria throughout the observation period.

The inclusion of a well-defined comparator group—recipients of kidneys from donors with normal histology—alongside standardized pathology review and comprehensive clinical screening enhance the robustness of these outcome comparisons in the present study. Persistent microscopic hematuria following transplantation did not result in adverse graft or recipient outcomes and was observed with similar frequency in both the TBMD and Normal Biopsy groups. This stands in apparent contrast to donor-focused data such as that presented by Kido et al. (19), which demonstrated that persistent glomerular hematuria in living donors is associated with an increased long-term risk of progressive post-nephrectomy kidney disease and proteinuria. It is critical to differentiate between these clinical contexts; while Kido et al. highlight potential long-term safety vulnerabilities within the native kidney setting of the donor, our data focuses on allograft performance inside the recipient. This dichotomy underscores the critical balance required during donor selection, where recipient benefit must be matched with lifetime clinical surveillance of the living donor.

The main limitation of this study is its retrospective design and the absence of long-term prospective donor data, which introduces the potential for selection and information biases. The median recipient follow-up duration provides only short- to intermediate-term insights, highlighting the need for extended surveillance to detect subtle, late-onset graft changes or chronic injury. Additionally, the lack of routine genetic testing during the historical study period precludes the complete exclusion of heterozygous COL4A mutations and related hereditary conditions. While genomic sequencing was available at the time, samples had to be sent to international laboratories abroad, resulting in prolonged turnaround times that were impractical for active transplant timelines. Consequently, our clinical protocol relied strictly on comprehensive clinical screening and tri-modal biopsy evaluation to guarantee safety. We also acknowledge that the isolated IMH group may feature an inherent histopathological heterogeneity, potentially including “late phenotype variants” or mosaic structural variants with undetected GBM abnormalities that were not captured by standard needle biopsy sample areas.

Recognizing the high proportion of first-degree familial donors within the Saudi Arabian demographic and the risks of consanguineous heritable traits, our center has recently updated its clinical protocol. We have successfully implemented in-house genetic testing capabilities, and frontline genetic characterization is now performed locally for all prospective living donors presenting with isolated microscopic hematuria. This technological advancement directly overcomes our historical logistical limitations, and ongoing prospective clinical follow-up of these genetically stratified donor cohorts is currently underway to further clarify late-onset risks. However, a major strength of this study remains its large cohort of recipients with TBMD and a well-defined comparative group with normal kidney biopsy findings.

In summary, findings from this relatively large recipient cohort demonstrate that kidney allografts from donors with biopsy-proven TBMD or isolated IMH with normal histology yield excellent outcomes. Recipients exhibited stable renal function and an absence of proteinuria throughout intermediate follow-up. Persistent microscopic hematuria, when present pretransplant, frequently persisted after transplantation but did not result in progressive graft dysfunction. These results support the judicious use of kidneys from carefully evaluated living donors with TBMD or IMH following comprehensive clinical and histopathologic assessment. Nevertheless, parallel long-term studies focusing on donor outcomes remain essential to confirm safety and to refine future donor selection criteria.

## Data Availability

The original contributions presented in the study are included in the article/Supplementary Material, further inquiries can be directed to the corresponding author.
